# Dihydroartemisinin-induced ferroptosis in acute myeloid leukemia: links to iron metabolism and metallothionein

**DOI:** 10.1038/s41420-023-01371-8

**Published:** 2023-03-17

**Authors:** E. Grignano, L. Cantero-Aguilar, Z. Tuerdi, T. Chabane, R. Vazquez, N. Johnson, J. Zerbit, J. Decroocq, R. Birsen, M. Fontenay, O. Kosmider, N. Chapuis, D. Bouscary

**Affiliations:** 1 INSERM U1016, Institut Cochin, Paris, France; 2 CNRS UMR8104, Paris, France; 3 Université Paris Descartes, Faculté de Médecine Sorbonne Paris Cité, Paris, France; 4 Equipe Labellisée Ligue Nationale Contre le Cancer (LNCC), Paris, France; 5 Assistance Publique-Hôpitaux de Paris, Hôpitaux Universitaires Paris Centre, Service d’Hématologie Clinique, Paris, France; 6 Assistance Publique-Hôpitaux de Paris, Hôpitaux Universitaires Paris Centre, Service d’Hématologie Biologique, Paris, France; 7 Assistance Publique-Hôpitaux de Paris, Hôpitaux Universitaires Paris Centre, Pharmacie, Paris, France; 8 Member of OPALE Carnot Institute, The Organization for Partnerships in Leukemia, Paris, France

**Keywords:** Cancer therapeutic resistance, Acute myeloid leukaemia

## Abstract

Artemisinin is an anti-malarial drug that has shown anticancer properties. Recently, ferroptosis was reported to be induced by dihydroartemisinin (DHA) and linked to iron increase. In the current study, we determined the effect of DHA in leukemic cell lines on ferroptosis induction and iron metabolism and the cytoprotective effect triggered in leukemic cells. We found that treatment of DHA induces early ferroptosis by promoting ferritinophagy and subsequent iron increase. Furthermore, our study demonstrated that DHA activated zinc metabolism signaling, especially the upregulation of metallothionein (MT). Supportingly, we showed that inhibition MT2A and MT1M isoforms enhanced DHA-induced ferroptosis. Finally, we demonstrated that DHA-induced ferroptosis alters glutathione pool, which is highly dependent on MTs-driven antioxidant response. Taken together, our study indicated that DHA activates ferritinophagy and subsequent ferroptosis in AML and that MTs are involved in glutathione regenerating and antioxidant response.

## Introduction

Acute myeloid leukemia (AML) denotes a group of highly heterogeneous diseases that all share a continuous activation of oncogenic signaling and an iron overload [1, 2]. Major breakthroughs in the treatment of these cancers have arisen in recent years leading to the emergence of targeted therapies such as midostaurin, venetoclax and IDH mutant inhibitors, and immunotherapies (Bispecific T Cells Engagers (BiTE) antibodies and CAR-T cells) [3]. Notably however, AML still has a poor overall prognosis and there remains an urgent need for new treatments.

Dihydroartemisinin (DHA), an extract derived from the Chinese plant *Artemisia annua* and an active derivative of arteminisinin (ART) [4], exerts cytotoxic effects against human malignancies derived from cancers of various origins, including breast, lung, colorectal, and lymphoma [5–9]. Recent studies have shown that the anticancer activity of ART derivatives including DHA are highly reliant on the iron-mediated cleavage of an endoperoxide bridge and subsequent ROS generation [10]. Since most cancer cells have higher iron uptake capacities than normal cells, DHA can exert selective toxicity toward tumors [5, 9, 11]. DHA modulates iron-related gene expression, and DHA toxicity is statistically associated with key regulators of iron levels such as transferrin receptor-1 (TFR1), ferritin light chain (FTL) or ceruloplasmin (CP) [12].

Ferroptosis refers to a caspase-independent non-apoptotic cell death mechanism that was first described by Dixon and colleagues in 2012 [13]. It is driven by the accumulation of lipid ROS, in which iron has a major role in catalyzing lipid peroxidation upon completion of the Fenton reaction. Hence, ferric iron favors lipid ROS generation and thereby induces ferroptosis. Ferroptosis has been extensively studied to date and several ferroptosis inducers have been described. Among these factors, compounds that decrease the glutathione (GSH) levels by inhibiting cysteine import [erastin [13] and sorafenib [14]], that inhibit the major antioxidant enzyme GPX4 [RSL3 [15], FINO2 [16], and FIN56 [17]] or directly chelate GSH [APR-246 [18]] can induce ferroptosis. By contrast, ferrostatin1 and liproxstatin are specific ferroptosis inhibitors, whereas iron-depleting agents like deferoxamine (DFO) or deferasirox (DFX) also impair this process [13].

DHA has been shown to induce ferroptosis in several cancer models [12, 19, 20], can downregulate SLC7A11 expression [21], and cooperates with standard chemotherapies to trigger ferroptosis [22]. Interestingly, DHA can also induce an autophagic degradation of ferritin, namely ferritinophagy [20], but also a lysosomal degradation of ferritin in an autophagy-independent manner, thus increasing the cellular free iron level and sensitizing the cell to ferroptotic death [23]. However, data on DHA ferroptotic effects in AML are scarce and its precise effect on iron metabolism and ferritinophagy needs to be clarified, given that this drug may constitute a valuable new therapeutic agent against this disease.

In our present study, we examined the effect of DHA on iron metabolism and ferritinophagy in a large panel of leukemic cells upon the induction of ferroptosis. We found in this analysis that DHA can upregulate the transcriptomic level of metallothionein (MT), a protein family involved in zinc metabolism. The inhibition of MT sensitizes the cells to ferroptosis which is induced by alleviating GSH regeneration and thus depleting this metabolite.

## Results

### An exogenous iron overload induces ferroptosis and sensitizes leukemic cells to ferroptosis induced by GSH-depleting compounds

**Fig. 1:**
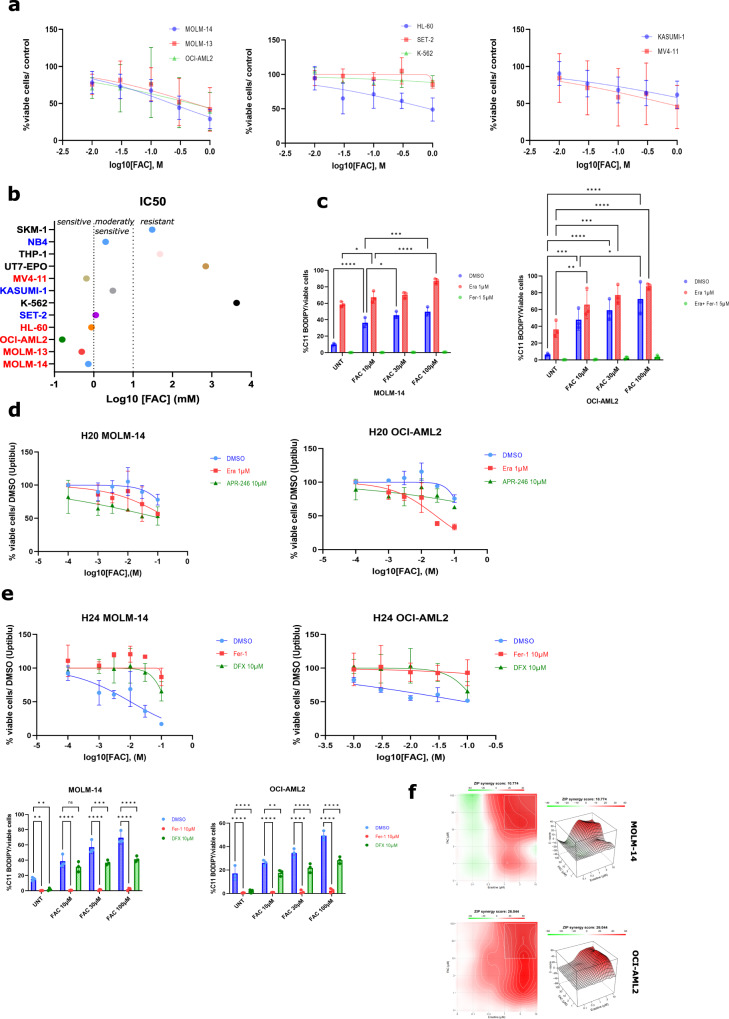
Ferric Citrate (FAC) induces ferroptosis in acute myeloid leukemia cells and sensitizes cells to ferrotopsis inducers toxicity. **a** Viability curves for the indicated cells at 24 h post treatment. Error bars ± standard deviation. **b** Half maximal inhibitory concentration percentage (IC_50_%) of FAC treatment for 24 h across a panel of AML cell lines based on cell viability (*n* = 3). In our subsequent experiments, we selected two AML cell lines sensitive to FAC in these concentration ranges. **c** C11-BODIPY staining of MOLM-14 cells treated with increasing doses of FAC + DMSO, FAC + erastine, FAC + Deferoxamine (DFO) or FAC + Ferrostatine-1 (Fer-1). Data are expressed in %positive cells (compared to unstained cells) (*n* = 3). **d** Viability curves for MOLM-14 or OCI-AML2 cells treated with FAC and various ferroptosis inducers at 24 h post treatment. Error bars, ± standard deviation. **e** Viability curves for MOLM-14 or OCI-AML2 cells treated with FAC and various ferroptosis inhibitors at 24 h post treatment. Error bars, ± standard deviation. **f** Illustrative synergy map of 24 h co-treatment of MOLM-14 cells with FAC and erastin. The mean cell viability of three independent experiments was used (*n* = 3).

In the absence of satisfactory data in the current literature on the addition of ferric iron or its modulation in leukemic models, we looked at the effects of an excess of exogenous ferric citrate iron (FAC) in AML on the onset of ferroptosis, as well as its effect on the sensitization to known ferroptosis inducers. To capture the heterogeneity of AML genetic abnormalities, we treated a large variety of AML cell lines with increasing concentrations of FAC, ranging from 10 to 1000 µM (Fig. 1a) and assessed cell viability after 24 h. We defined three groups in terms of sensitivity to FAC treatment according to the IC_50_ value i.e., sensitive cells whose IC_50_ was <1000 µM (in red), resistant cells whose IC_50_ was >2000 µM (black), and moderately sensitive AML cell lines whose IC50 was between 1000 µM and 2000 µM (Fig. 1b). The most sensitive AML cell line and the most resistant among this panel were OCI-AML2 (IC_50_ = 157 µM) and K562 (IC_50_ = 4261 µM), respectively. We used the two most sensitive cell lines for all of our subsequent experiments, ie MOLM-14 and OCI-AML2. We then confirmed that FAC toxicity was related to ferroptosis induction using C11-BODIPY specific staining, and that FAC could sensitize leukemic cells to ferroptosis induced by erastin (Era) (Fig. 1c). We observed from this analysis that FAC could sensitize MOLM-14 and OCI-AML2 leukemic cells to ferroptotic cell death induced by low doses of erastin (1 µM) or APR-246 (10 µM) agents acting on the pool of intracellular GSH (Fig. 1d), and that the effect of FAC was reversed by the ferroptosis inhibitor ferrostatin-1 (Fer-1) or by iron chelators such asdeferasirox (DFX) (Fig. 1e). To assess the synergistic effect between two compounds, we used a ZIP model score, a score based on the observed drug combination responses compared with expected combination responses calculated by means of synergy scoring model. Consequently, the drug combination is either classified as synergistic when the ZIP score is positive (i.e., combination effect is higher than expected) or antagonistic when the ZIP score is negative (i.e. combination effect is lower than expected). [24] According to ZIP model score, FAC and erastin exhibited a synergistic antileukemic effect (Fig. 1f). Collectively, these data indicates that an exogenous iron overload alone can trigger ferroptosis. Moreover, combination of iron overload with erastin further sensitizes certain AML cells to the ferroptotic cell death.

### DHA induces early death by ferroptosis

**Fig. 2:**
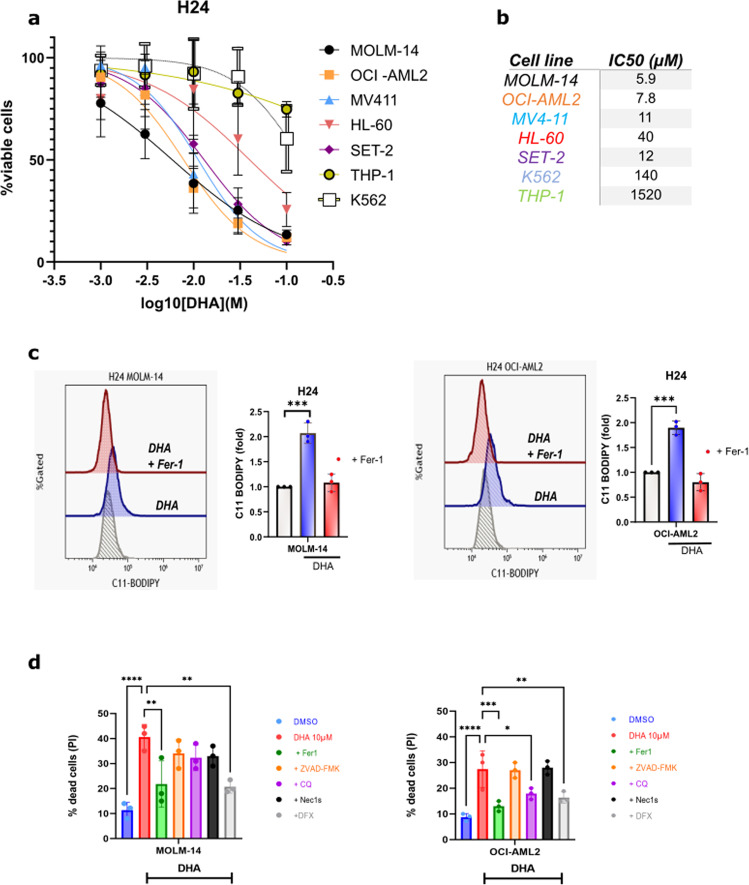
DHA induces early ferroptosis in AML cells. **a** Viability curves for the indicated cells at 24 h post treatment. Error bars, ± standard deviation (*n* = 6). **b** IC_50_% of DHA treatment for 24 h across the same panel of AML cells based on cell viability (*n* = 6). **c** C11-BODIPY staining of MOLM-14 and OCI-AML2 cells treated with increasing with DHA and DHA + Fer-1 Data are expressed in mean fluorescence intensity (MFI) (fold/ DMSO-treated cells) (*n* = 3). **d** Apoptosis % (PI staining) of MOLM-14 and OCI-AML2 cells treated with DHA and various cell death inhibitors at 24 h and 48 h (*n* = 3).

We next sought to modulate intracellular iron stores in a more fine-tuned way via ferritinophagy. Indeed, this recently described phenomenon seemed opportune as it can be triggered from a pre-existing ferritin stock, a situation frequently found in AML patients. As no compound has yet been found that can specifically mimic the effects of ferritinophagy, we decided to assess whether DHA could induce this process since it has been described as one of its mechanisms of action. We used a panel of the same AML cell lines used in Fig. 1A from the three categories of FAC sensitivity and exposed them for 24 h to increasing concentrations of DHA (Fig. 2a). Interestingly, the same AML cell lines that were sensitive to FAC-induced ferroptosis were also sensitive to the dose-dependent antileukemic effects of DHA, except for the SET2 cell line that was moderately sensitive to FAC but highly sensitive to DHA (Fig. 2a, b). Regarding DHA sensitivity, the MOLM-14 and OCI-AML2 cell lines were again found to be the most sensitive and were subsequently selected for further analysis. Using C11-BODIPY staining, we confirmed that DHA triggered strong ferroptosis which was reversed by Fer-1 co-treatment, as expected (Fig. 2c). We next sought to verify if ferroptosis was the main cell death mechanism induced by DHA in these leukemic cells. We preincubated AML cells with either Fer-1, ZVAD-FMK (a pan caspase inhibitor), chloroquine (a late autophagy inhibitor), necrostatin-1s (Nec1s) or DFX, a potent iron chelator and treated them for 24 h with 10 µM of DHA. The specific ferroptosis inhibitors Fer-1 and DFX were the only ones to strongly inhibit DHA-mediated cell death in both AML cell lines (other than slight effects of chloroquine in OCI-AML2 cells) after 24 h of treatment, whereas caspase inhibition could not reverse the cell death effect of DHA (Fig. 2d). Collectively, these data indicated that DHA induces an early ferroptotic cell death response in AML cell lines that are sensitive to FAC.

### DHA cooperates with FAC to increase the intracellular iron pool

**Fig. 3:**
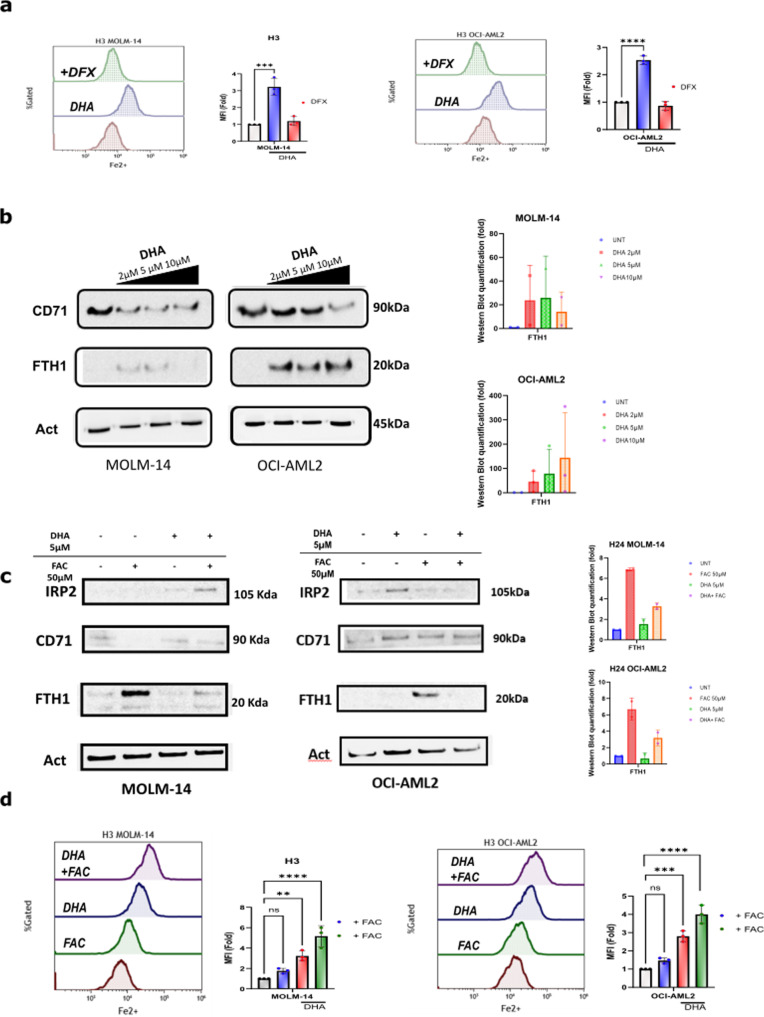
DHA increases intracellular iron and synergizes with low dose FAC treatment. **a** Fe2+ level assessed by Sirhonox straining after DH A or DHA + DFX treatment for 3 h in MOLM-14 or OCI-AML2. Data are expressed in in MFI (fold/DMSO-treated cells) (*n* = 3). **b** Immunoblotting analysis of CD71 and FTH1 in MOLM-14 and OCI-AML2 cells treated at increasing doses of DHA (1, 5 and 10 µM) for 24 h. **c** Immunoblotting analysis of IRP2, CD71 and FTH1 in MOLM-14 cells treated with FAC 10 µM, DHA 5 µM or both for 24 h. **d** Fe2+ level assessed by Sirhonox staining after DHA, FAC treatment or combination of both for 3 h. Data are expressed in mean fluorescence intensity (MFI) (fold/ DMSO treated cells) (*n* = 3).

To study the effects of DHA on iron metabolism, we treated MOLM-14 and OCI-AML2 with 5 µM DHA for different time periods and assessed the labile iron pool (LIP) by FC using a specific Fe2+ dye (FerroFarRed) that stains Fe2+ in the endoplasmic reticulum and Golgi apparatus. We assumed that this staining intensity reflected the intracellular LIP level. We observed a rapid 2 to 3-fold increase in the LIP amount after DHA treatment in both AML cell lines, which was reversed by a co-treatment with DFX (Fig. 3a). This increase led to a dose-dependent counter downregulation of transferrin receptor-1 (TFR1, also known CD71) and subsequent increase in the FTH1 level after 6 h of treatment, despite no major FTH1 accumulation between DHA doses of 2 and 10 µM in MOLM-14, as analyzed by western blot (Fig. 3b) *(See uncropped WesternBlots in supplemental)*. The counter downregulation of CD71 level expression after 24 h of DHA exposure was also assessed by FC analysis in MOLM-14 and OCI-AML2 cells (***S1***). We then determined if co-treatment with DHA and FAC could cooperate in increasing the LIP and the iron-mediated toxicity in MOLM-14 and OCI-AML2 cells. While FAC treatment (10 µM) for 24 h alone increased FTH1 iron storage and led to a drastic CD71 downregulation, co-treatment with DHA (5 µM) for 24 h induced an IRP2 response characterized by a FTH1 expression decrease (iron release) and a slight CD71 expression upregulation (Fig. 3c). By assessing the LIP concentration with the FerroFarRed dye, we observed a rapid cooperative effect on LIP increase (4 to 5-fold) with FAC and DHA co-treatment in both AML cell lines (Fig. 3d).

Collectively, these data indicated that DHA induces a rapid and important increase in LIP in sensitive AML cell lines. FAC co-treatment further enhanced the iron- accumulation.

### DHA-induced ferritinophagy participates in its toxicity

**Fig. 4:**
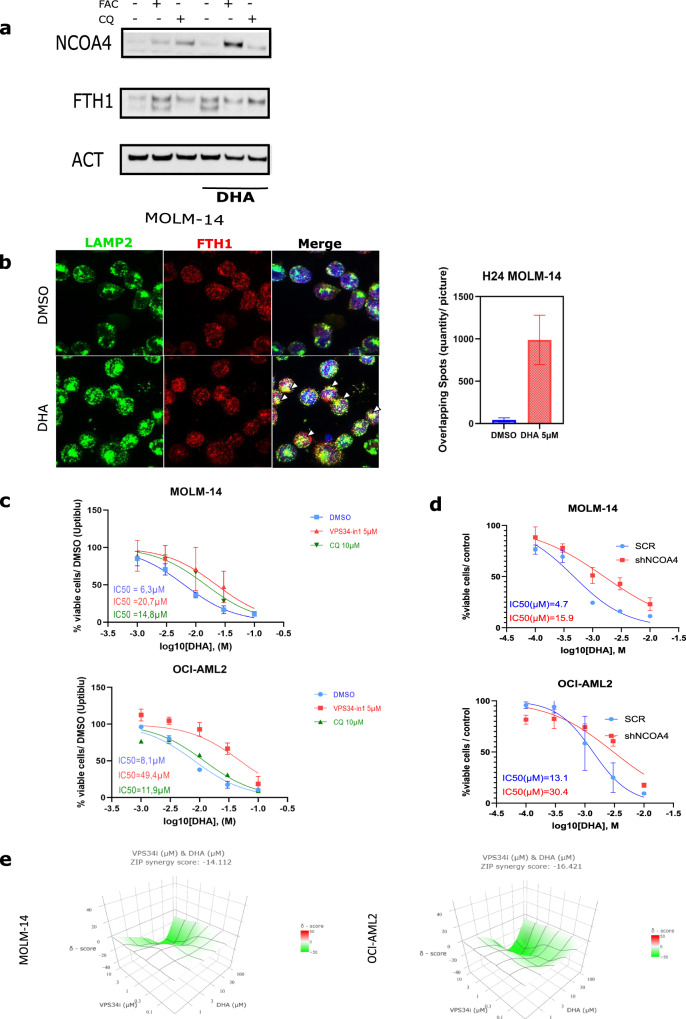
DHA induces ferritinophagy in AML cells. **a** Immunoblotting analysis of LC3, NCOA4 and FTH1 in MOLM-14 cells treated with, FAC 10 µM, CQ 10 µM, DHA 5 µM or combination of FAC + DHA or CQ + DHA for 12 h. **b** Immunofluorescence analysis of FTH1 or LAMP2 in MOLM-14 treated with DHA 5 µM, FAC 10 µM or both for 16 h. **c** Viability curves for the indicated cells at 24 h post treatment with DMSO, chloroquine (10 µM) or VPS-34in1 (5 µM) and DHA at increasing doses. Error bars, ± standard deviation (*n* = 3). **d** Immunoblotting analysis of CD71, NCOA4, and FTH1 in MOLM-14 and OCI-AML2 cells treated with, FAC 10 µM, VPS34-In1 5 µM, and DHA 5 µM or combination of FAC + DHA or VPS34-In1 + DHA for 24 h. **e** Viability curves for MOLM-14 or OCI-AML2 cells transduced with scramble or shNCOA4 treated with DHA at increasing doses for 24 h and corresponding IC_50_%. Error bars, ± standard deviation (*n* = 3).

We sought to confirm that DHA could induce ferritinophagy in our AML model. We first assessed the effect of CQ (10 µM) or FAC (50 µM), in addition to DHA (5 µM), for 24 h in the MOLM-14 cell line. Whereas CQ leads to an accumulation of NCOA4 expression and the fading of the FTH1 lysosomal band (lower band), DHA alone increased FTH1 expression. Surprisingly, FAC addition to DHA halted the ferritinophagy flux (increase in the upper band and fading of the lower band), thereby mimicking CQ addition (Fig. 4a). To address the question of whether the FTH1 increase was caused by NRF2 activation after DHA treatment, we analyzed the expression of NRF2 after DHA exposure (5 µM) for 6 h, alone or with the NRF2 inhibitor brusatol (10 nM). We observed that increased DHA-mediated FTH1 expression and xCT was linked to NRF2 antioxidant response (***S.2a***). We next performed an immunofluorescence experiment using antibodies against LAMP2 (green) and FTH1 (red) after exposure to DHA (5 µM) for 16 h. We confirmed that DHA-induced the colocalization between FTH1 and the lysosomal marker LAMP2 (white arrows) (Fig. 4b). To further study the impact of ferritinophagy on DHA-induced toxicity, we treated the MOLM-14 or OCI-AML2 cells with the more specific ferritinophagy inhibitor, VPS34-in1, that specifically inhibits VPS34 catalytic activity (5 µM) and compared it to CQ (10 µM) co-treatment upon DHA dose increases. VPS34-in1 more strongly reduced the DHA IC_50_ than CQ (see Fig. 4c and ***S2b*** for VPS34-in1 effects on FTH1 and NCOA4 level, determined by western blot, compared to FAC or the lysosomal inhibitor BafA1). By using a synergistic model, we confirmed that both DHA and VPS34-in1 displayed a strong antagonist effect (ZIP score < 10) (Fig. 4e). We then assessed if NCOA4 genetic inhibition could mitigate DHA-induced toxicity. After assessing an NCOA4 effective knockdown and FTH1 increase in MOLM-14 and OCI-AML2 cells with two different inducible NCOA4 shRNA constructs (***S 2c***), we compared the DHA treatment effects in the shSCR (scrambled) or shRNA NCOA4 AML cell lines. We observed an IC_50_ increase in the shRNA NCOA4 transduced MOLM-14 and OCI-AML2 cell lines treated with DHA, as expected (Fig. 4d). Overall, these results indicate that antileukemic activity of DHA is related to ferritinophagy induced by DHA.

### Transcriptomic analysis identification of the upregulation of MTs upon DHA treatment

**Fig. 5:**
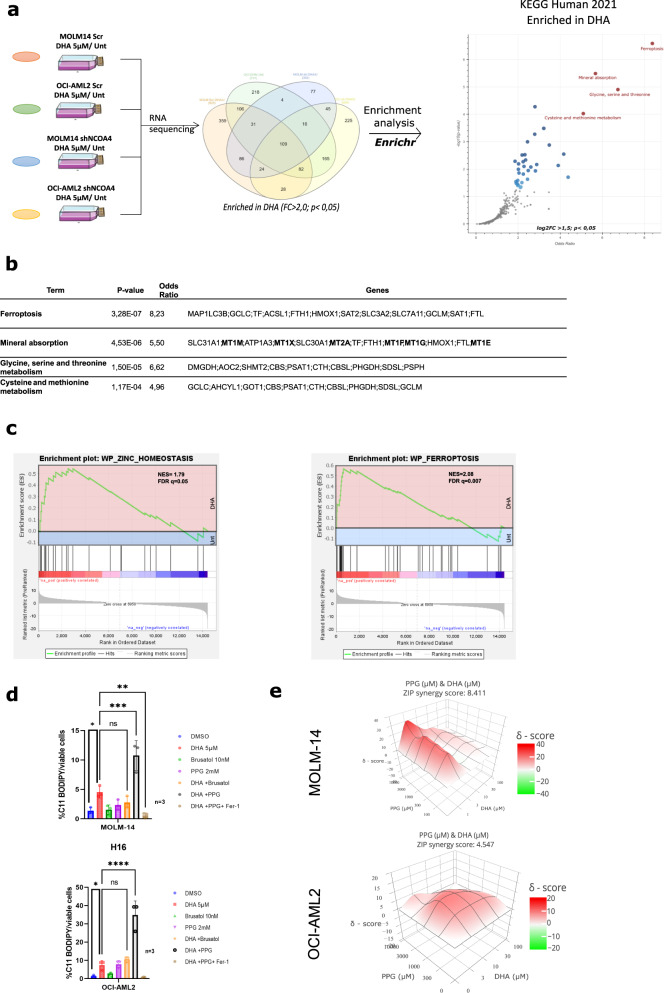
Transcriptomic analysis identifies Zinc metabolism and metallothionein as DHA toxicity modulators. **a** Left: experimental design: MOLM-14 and OCI-AML2 transduced with scramble or shNCOA4 were treated with DMSO or DHA 5 µM for 24 h. RNA was extracted and transcriptomic analysis was performed on triplicates. Middle; Venn diagram of the RNA enriched in DHA group (*p* < 0.05). Right: pathways enriched in the DHA group according to Enrichr analysis (*p* < 0.05) (In red: the top-4 pathways enriched). **b** Transcriptomic data were processed in GSEA enrichment analysis according to KEGG2022 pathway. Ferroptosis and Zinc homeostasis pathway enrichment are shown. **c** Table of the top-4 pathways enriched in Enrichr analysis and the corresponding genes involved. **d** C11-BODIPY staining of OCI-AML2 cells treated DHA 5 µM, Brusatol 10 nM, PPG 2 mM or combination of DHA + Brusatol or DHA + PPG-1. Data are expressed in %positive cells (compared to unstained cells) (*n* = 3). **e** Illustrative synergy map of 24 h co-treatment of OCI-AML2 cells with DHA and PPG. The mean cell viability of three independent experiments was used (*n* = 3).

We wished to elucidate the mechanisms involved in the cytoprotective effects against DHA antileukemic activity in AML. We performed RNAseq analysis on the MOLM-14 and OCI-AML2 cells, expressing either a shSCR or a shRNA NCOA4, after treatment with DHA at 5 µM for 24 h. Raw data of the most up- and downregulated genes in the MOLM-14 shSCR and OCI-AML2 shSCR cells are presented in ***S3***, but none of these genes were of interest from our perspective. As shown in the Venn diagram in Fig. 5a, 109 genes were common to the four groups and were upregulated after DHA treatment in both AML cell lines (FC > 2.0; *P* < 0.05). We next performed enrichment analysis using the Enrichr webtool by pooling the four groups after DHA treatment. According to KEGG Pathway 2021,the ferroptosis pathway which involves the genes involved in iron metabolism (FTH1, FTL, TF, HMOX1), genes involved in amino acid transport and metabolism (SLC7A11, SLC3A2, GCLC, GCLM) and genes involved in polyamine metabolism (SAT1, SAT2), was found in this analysis to be the most enriched (OR = 8.23; *P* < 0.001) (Fig. 5b). Cysteine and serine-threonine metabolism were also among the top score enriched pathways, as expected. The differential expression of the main proteins between cells treated with DHA, and control cells, for ferroptosis, iron metabolism and NRF2 pathways are shown in ***S4***. Surprisingly, upon DHA treatment, zinc metabolism pathway is significantly enriched in the MOLM-14 and OCI-AML2 lines (OR = 5.5; *P* < 0.001). This pathway comprises several MT members (MT1M, MT1X, MT2A, MT1F, MT1G, MT1E), zinc (SLC30A1) or copper transporter (SLC31A1). We confirmed these enrichment pathways by using GSEA analysis and again identified ferroptosis (NES = 2.08; *P* < 0.01) and zinc metabolism (NES = 1.79; *P* < 0.05) in the top score pathways enriched upon DHA treatment (Fig. 5c). The data concerning the six-top score pathways in either MOLM-14 SCR or OCI-AML2 SCR are presented in ***S5***. Notably, results showed that after DHA treatment, the NRF2 antioxidant response pathway is statistically activated (*P* < 0.05) in both cell lines, confirming its previously mentioned role. We confirmed the upregulation of the six MT isoforms identified in enrichment analysis by qPCR in MOLM-14 and OCI-AML2 after DHA treatment (5 µM) for 24 h (***S6a***). The analysis of MT isoforms by qPCR in the less sensitive leukemic cell lines THP-1 and K562 showed a moderate upregulation in response to DHA treatment, up to 2-fold vs 3 to 6 fold in MOLM-14 and OCI-AML2 respectively (***S6b***). To confirm the involvement of MT upregulation in the antioxidant response after DHA treatment, we used the PPG compound that has been shown to downregulate all MT isoforms [25]. PPG (2 mM) co-treatment with DHA in MOLM-14 and OCI-AML2 cells led to a very strong increase in ferroptosis as demonstrated by C11-BODIPY staining, whereas co-treatment with the NRF2 inhibitor brusatol (10 nM) produced no effect (Fig. 5d). We aimed to assess MT inhibition by PPG in the same aforementioned less sensitive leukemic cell lines (i.e., THP-1 and K562) but couldn’t observe the same effect compared to MOLM-14 or OCI-AM2 (***S6c***)*.* Finally, we performed a synergistic assay between DHA and PPG in the MOLM-14 and OCI-AML2 lines and found that both compounds exhibited a moderate synergistic effect (Fig. 5e). Collectively, these data confirm that MT is upregulated in response to DHA treatment and that MT pan-inhibition with PPG sensitize leukemic cells to DHA-induced ferroptosis.

### Specific MT isoform downregulation sensitizes AML cells to ferroptosis inducers by mitigating the GSH pool

**Fig. 6:**
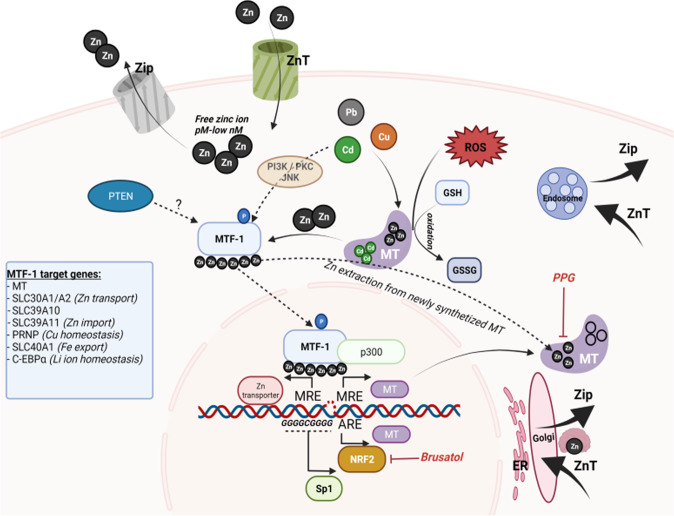
Illustrative draw of the Metallothionein regulation and the involvement of MTF1 and NRF2 regulation. The unique chemical properties described above determine the key roles of MTs in cellular Zn++ homeostasis by low affinity binding of ~5–15% of the cell’s Zn ion pool in combination with two classes families of Zn transporters, namely, Zrt- and Irt-like proteins (ZIP, transporting Zn++ into the cytoplasm) and Zn transporters (ZnT, transporting Zn++ away from the cytoplasm). One MT can contain up to 7 atoms of Zn2+. MT are involved in heavy metals detoxification, like Cu2+, Cd+ or Pb+. For instance, MT oligomerization can occur either in native/non-oxidative forms or in oxidative forms induced by high Cd2+ concentrations, where MT subunits are covalently linked via disulfide bridges. Under oxidative stress, MT can release Zn2+ (apo-MT) to form thiol groups and participates in GSSG/ GSH reduction. Basal TM activity is regulated by several general transcription factors. Additionally, MT can be activated by a variety of stimuli, including metal ions, cytokines, and growth factors. Several inducible expression regulators of MT genes have been identified, including metal-responsive element (MRE). MREs are classically required for induction by metals. MREs act in conjunction with the zinc-dependent and zinc-responsive transcription factor MTF1 which is involved in the inducible expression of MTs. In this sense, MREs, MTF1 and the essential Zn ions that associate with MTF1, contribute to the basal and inducible expression of MTs. MTF1 also independently facilitates the recruitment of Sp1 and p300 to the protein complex in response to zinc. Along with MREs, many MT promoters contain an ARE consensus sequence, and are induced by NRF2 mediated antioxidant response. All promoters of the MT gene also contain at least one GC box (consensus sequence GGGGCGGGG) that responds to members of the constitutive Sp/XKLF zinc finger transcription factor family, including Sp1. Finally, MTF1 has been shown to be constitutively phosphorylated at serine and tyrosine residues under basal conditions in both human and mouse cells. Induction with transition metals (both cadmium and zinc) increases the level of MTF1 phosphorylation through a complex pathway involving protein kinase C (PKC), phosphoinositol-3 kinase (PI3K), c-jun N-terminal kinase (JNK), tyrosine kinase, casein kinase II, and calcium signaling, as well as PTEN, which can interact with MTF1 via its phosphatase/ C2 domain.

MTs are essential in zinc metabolism, heavy metal detoxification and in antioxidant defenses by acting on GSH regeneration. Metal-responsive transcription factor-1 (MTF1) plays a key role in the transcription of MT after oxidative stress is produced by a heavy metal overload. The MT genes contain a Metal Response Element (MRE) in their promoters that is responsive to MTF1 binding and antioxidant response elements (AREs) which facilitate their upregulation in response to NRF2 activation. The essential components of MT regulation are shown in Fig. 6. We first hypothesized that NRF2 activation could drive MT upregulation upon DHA treatment and constructed two shRNAs directed against NRF2 (***S7****,* sh2 NRF2 was chosen for its better efficacy). However, we did not observe MT downregulation after NRF2 inhibition (***S8***). We then decided to inhibit MT2A directly, as it was found to be the most upregulated isoform in our transcriptomic analysis after DHA exposure. This MT2A genetic inhibition produced no effect on DHA sensitization and a slight effect after treatment with ferroptosis inducers acting on the GSH pool, such as erastin, APR-246 or FIN56 (***S9***). To decipher this lack of an effect, we assessed the RNA level of the MT isoforms by qPCR after DHA treatment. MT2A genetic inhibition in the context of DHA treatment led to a rapid counter upregulation of other MT isoforms, mostly MT1M (***S10***). Despite this feedback regulation, MT2A inhibition was able to sensitize MOLM-14 and OCI-AML2 cells to erastin treatment with an increase in in the GSSG (oxidized form)/ GSH ratio (***S11***).

**Fig. 7:**
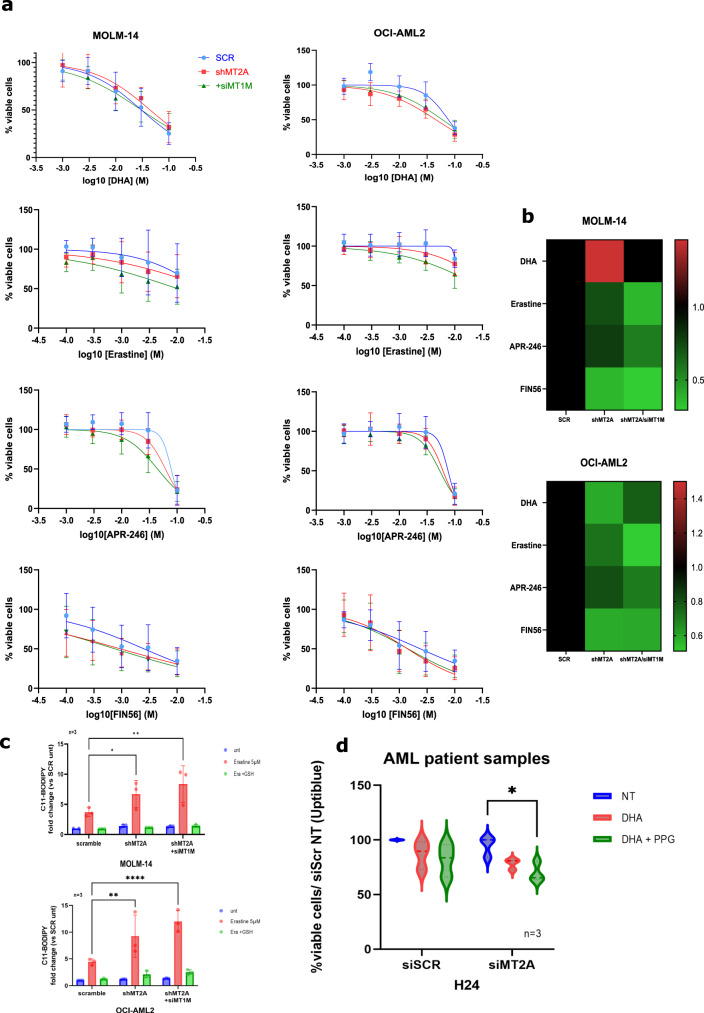
MT2A and MT1M genetic invalidation sensitizes cells to ferroptosis inducers. **a** Viability curves for the indicated cells at 24 h post treatment. Error bars ± standard deviation are shown (*n* = 6). **b** Heat maps showing IC_50_% of the indicated compounds after 24 h of exposure in MOLM-14 and OCI-AML2 cells transduced with shSCR, shMT2A or shMT2A and siMT1M for 48 h (*n* = 6). **c** C11-BODIPY staining of MOLM-14 and OCI-AML2 cells transduced with shSCR, shMT2A or shMT2A and siMT1M for 48 h and treated with DMSO, erastine (5 µM) or erastine + GSH (1 mM). Data are expressed in fold change compared to SCR non treated cells (*n* = 3). **d** Violin plots representing the viability in Uptiblue assay for three AML patients transduced with siSCR or siMT2A for 48 h and treated with DHA (50 µM) alone or DHA + PPG (2 mM) for 24 h.

We therefore decided to co-inhibit MT2A along with the MT1M isoform by using a siRNA directed against MT1M in the shRNA MT2A-transduced MOLM-14 and OCI-AML2 AML lines. The inhibition of both isoforms led to a slightly increased sensitization to cell death upon DHA treatment for 24 h (10 µM). More importantly, the inhibition of both isoforms sensitized these cells to the death induced by ferroptosis inducers acting on the GSH pool (erastin, APR-246 or FIN56) (Fig. 7a). Notably, the IC_50_ was halved for erastin toxicity in the MOLM-14 and OCI-AML2 cells (Fig. 7b). Finally, we wanted to confirm that this effect was mediated by a GSH regeneration impairment and linked to ferroptosis induction. We treated shMT2A or shMT2A + siMT1M transduced MOLM-14 or OCI-AML2 cell lines with erastin (5 µM) or erastin + GSH (1 mM) for 16 h. These double transduced cell lines showed a significant increase in C11-BODIPY staining compared to single shMT2A or SCR cells, and this effect was fully reversed upon GSH co-treatment (Fig. 7c). We then transfected samples of primary medullar leukemic cells from three patients (see details in Supplemental figure legends) with Cya3-tagged siMT2A for 48 h to control for transfection efficacy and treated them with DHA (50 µM) or DHA + PPG (2 mM). We observed an increase in cytotoxicity with both compounds in siMT2A-transfected cells compared to DHA alone (Fig. 7d). Collectively, these data supported a protective role of MT expression through the generation of an anti-ferroptotic response to DHA in AML cells that, in effect, is mediated by their role in regenerating the GSH pool. Our current findings also suggest that MT chemical inhibition can cooperate with DHA in primary AML cells in patients.

## Discussion

Ferroptosis is quite distinct from other types of regulated cell death such as apoptosis and necroptosis. It is characterized by the excessive accumulation of ROS from iron metabolism and lipid peroxidation [26]. Increasing evidence has now revealed that inducing ferroptosis could constitute an attractive therapeutic strategy to overcome cancer cell resistance to drug-induced apoptosis [27].

Previous studies have demonstrated that DHA promotes ferroptosis through autophagy induction and more specifically through ferritinophagy [20], even if conflicting results have emerged recently [28]. In a previous study, Du and colleagues found that DHA can trigger ferroptosis in the HL-60 leukemic cell line by upregulating the activity of the AMPK/mTOR/p70S6k signaling axis, and through subsequent autophagy induction, mitochondrial dysfunction and iron release [20]. Herein, we found that DHA could effectively trigger ferroptosis in different leukemic cell lines and observed its toxicity independently of the genetic landscape. We further confirmed that DHA induces an early ferritinophagic flux, thus promoting iron release and enabling lipid peroxidation, and that DHA toxicity may be strengthened by exogenous ferric iron addition at low doses. Paradoxically, high doses of ferric iron impaired DHA-induced ferroptosis, probably due to the enhanced-iron storage, which is induced by immediate stimulation of FTH1 synthesis.

Our present study also highlights a novel role of zinc metabolism, especially the MT family of small ubiquitous proteins involved in heavy metal detoxification and the response to oxidative stress, in the regulation of ferroptosis. To our knowledge, the only previous study mentioning a role of MT in the context of ferroptosis used an hepatocarcinoma model, where an MT1G isoform turned out to mitigate sorafenib-induced ferroptosis through NRF2 pathway activation [29]. In our current study, we observed a global upregulation of some MT isoforms, predominantly MT2A, MT1M, MT1X, MT1E, and MT1F, under DHA exposure whereas the NRF2 pathway activation was not involved. This overall upregulation complexifies the modulation of MT, as the inhibition of one isoform appears to be immediately compensated for by the upregulation of others in the context of DHA treatment. Nevertheless, the use of a pan irreversible chemical inhibitor of MT isoforms like PPG may overcome this limitation [25, 29].

While the inhibition of the most overexpressed isoforms in our current experiments, MT2A and MT1M, seems not to play a major role on the cytotoxic response of ARTs, its impact was found to be preponderant in the response ferroptosis induction by compounds modulating the GSH pool, either via the inhibition of the intracellular import of cysteine (erastin), direct GSH inhibition (APR-246) [18], or an indirect block of the GPX4 enzyme (FIN56). Previous studies have revealed that MT may regulate the depletion of GSH through the modulation of its biosynthetic enzymes [30]. Herein, we confirmed that MT inhibition impairs GSH regeneration and subsequent GPX4 activity, thus sensitizing cells to ferroptotic cell death. The possibility is not excluded that MT inhibition may also modulate the labile iron pool as previous studies have demonstrated the putative capacity of MT to link Fe2+ in vitro [31].

Several completed clinical trials (NCT00764036, NCT02353026, NCT02354534, NCT03100045, NCT02304289) and ongoing clinical trials (NCT02633098, NCT03093129, NCT03792516, NCT04098744, NCT02786589) have reported on the efficiency and acceptable tolerance of artemisinins in cancer patients. Among other cell death inducers, DHA can specifically trigger early ferroptosis in leukemic cells and exhibit a synergistic effect with MT inhibition to promote lipid peroxidation. Nonetheless, ferroptosis inducers for which GSH depletion constitutes the main mechanism, e.g., erastin, APR-246 or FIN56, may be better candidates to foster ferroptotic cell death in the context of MT inhibition. Hence, our present study provides evidence that MT modulation may be a promising adjuvant that will improve the ferroptosis response in leukemic cells in patients. We also here provide a paradigm for a more in-depth understanding of ferroptosis as a potential future cancer therapy.

## Conclusion

DHA can trigger ferroptosis in different leukemic cells of varying genetic background, and this ferroptotic cell death is mediated mostly, but not exclusively, by ferritinophagy induction and subsequent intracellular iron release. Furthermore, MT upregulation has been shown for the first time to be involved in ferroptosis mitigation, especially in the context of GSH-depleting compounds. Subsequent MT inhibition may sensitize leukemic cells to lipid peroxidation in vitro by impairing GSH regeneration. This strategy requires further investigation to be potentially translated to a viable in vivo clinical application.

## Material and methods

### Cell lines and reagents

MOLM-14, OCI-AML2, HL-60, SET2, MV4-11, K562, THP-1, UT7-EPO, SKM1, NB4 and KASUMI-1 AML cell lines were used. Patients provided written informed consent in accordance with the Declaration of Helsinki. Bone marrow (BM) samples were obtained from five patients with newly diagnosed AML (characteristics provided in the Supplementary Table S1). Cells were cultured in RPMI with glutamine (Gibco61870, Life Technologies® Saint Aubin, France) supplemented with 10% fetal bovine serum (FCS) and 4 mM glutamine. Ferric Citrate (FAC) was purchased from Sigma. DHA, Ferrostatin-1, Deferoxamin (DFO), Deferasirox (DFX), QVD-OPH, APR-246, VPS34-in1, erastin, FIN56, and RSL3 for the in vitro study were sourced from Selleckchem (Houston, TX). L -C-Propargylglycine (PPG), Chloroquine and doxycycline were obtained from Sigma–Aldrich (Saint-Louis, MO). FINO2 was purchased from Cayman Chemicals (Ann Arbor, MI).

### Constructs

Inducible short hairpin RNA (shRNA) targeting NCOA4, MT2A or NRF2 were constructed and purchased from Eurofins using the following sequences: NCOA4 #1: GGCAGCTCCAGTAATAGAGAA; #2 GGTTATCAAGCTCCTTACATA; NRF2 #1 GCTCCTACTGTGATGTGAAAT; #2 GCACCTTATATCTCGAAGTTT MT2A #1 GCAAATGCAAAGAGTGCAAAT; #2 CAAATGCACCTCCTGCAAGAA and cloned into a pLKO-Tet-On–inducible or pLKO.1 plasmid (plasmids #21915 [32]; Invitrogen, Cambridge, MA). siRNA-mediated silencing of gene expression was performed with Lipofectamine RNAimax (Thermofischer, #13778150) according to the manufacturer’s guidelines. MT1M (5’->3’#1 GCA AAG GGA CGU UGG AGA ACU; #2UGU GUC UGC AAA GGG ACG UUG) or MTF1(#1 CCU CAC CUU UCA GAC GUA UUU; #2 GCU CUC UUA CAA CCU CCA GAA) or scramble siRNA constructs were designed and ordered from Eurofins with modified 5’ Cy5 reporter to control transfection efficacy.

### Lentiviral packaging and transduction

293-T packaging cells were used to produce lentiviral constructs through co-transfection with plasmids encoding lentiviral proteins. After 24 h and 48 h of transfection, supernatants were collected and ultracentrifuged at 22,000 rpm for 90 min at 4 °C over 2 consecutive days, filtered through a 0.4 μm filter and subsequently stored at −80 °C. AML cell lines were seeded at 2 × 10^6^/mL and 2–10 mL aliquots of lentiviral supernatants were added for 3 h. Cells were then grown in 10% fetal calf serum medium for 24 h and further selected with puromycin for 48 h. For shRNA induction, 100 mg/mL doxycycline was added to the culture medium for 48 or 72 h.

### Flow cytometry-based assay

Data acquisition and data analysis were conducted at the Cochin Cytometry and Immunobiology Facility. For iron assay measurements using BioTracker Far-red Labile Fe2+ Dye (called FerroFarRed, #**SCT037**, Sigma; Thermofischer; Waltham, MA), 2 × 10^5^ cells were washed twice in PBS to remove extracellular Fe2+ then labeled with 2 µM Sirhonox for 60 min in a tissue culture incubator (37 °C, 5% CO^2^) in the dark and then washed twice again. The pelleted cells were then resuspended in 0.3 mL of PBS. FCM data were collected using a BD Navios flow cytometer. 10,000 events were recorded for analysis. Data analysis was then carried out with KALUZA software. For lipid peroxide production measurements using C11- BODIPY (581/591) (2 mM) (Thermofischer, Waltham, MA), 2 × 10^5^ cells were labeled with C11-BODIPY in 1 mL of warm complete medium for 10 min in a tissue culture incubator (37 °C, 5% CO^2^) in the dark. Cells were then washed twice and resuspended in 200 µL of fresh PBS. FCM data were collected using a C6 Accuri flow cytometer (Becton Dickinson, Le Pont de Claix, France) with CFlow Plus software. 10,000 events were captured for subsequent analysis with CFlow Plus software (Becton Dickinson, Le Pont de Claix, France).

### GSSG/GSH assay

For GSSG/GSH ratio measurements using colorimetric GSH assay kit (Abcam), the protocol from the manufacturer was used. Briefly 2 × 10^5^ cells per condition were seeded, cells were centrifuged and resuspended in cold PBS and then lysed in ice-cold GSH buffer. A solution mix containing NADPH mix, GSH reductase and GSH reaction buffer was prepared. 20 µL of each sample or GSH standard solution to 160 µL of the reaction mix and incubated for 10 min at RT. Finally, the absorbance at 405 nm or 415 nm was assayed using a Typhoon FLA9500 microplate reader (GE Healthcare; IL).

### Immunofluorescence

Cells were plated on Polylysine coating slides, treated for 16 h with DHA 5 µM ± FAC 10 µM. Cells were then fixated with methanol, saturated with PBS/BSA 3% and incubated with anti-FTH1 and anti-LAMP2 antibody overnight. After three washes with PBS–0.2%BSA-tween 0,01%, cells were incubated with secondary antibodies, washed three times before mounting on cover slides with Invitrogen™ Fluoromount-G™ Mounting Medium, with DAPI. Images were collected with 16 z-series optical sections collected with a step size of 0.2 µm, using Inverted microscope Leica DMI6000 Spinning Disk. Z-series are displayed as maximum z-projections.

### RNA extraction, QPCR and transcriptomic analysis

Total RNA isolation were perf,ormed using RNA extraction kit (Qiagen). 1000 ng of each sample was then reverse transcripted using RT Maxima (ThermoFisher) and cDNA from samples were amplified using specific primers: MT2A 5’-CTCCAAGTCCCAGCGAAC-3’; MT1A5’-GCTTGAGATCTCCAGCCTTACC-3’; MT1X5’-TCTCCTTGCCTCGAAATGGAC-3’; MT1M5’-GCTTGAGATCTCCAGCCTTACC-3’; MT reverse 3’-TTGCAGGAGGTGCATTTG-5’; The data were normalized to actin RNA (human Actine 5′-GGGTCAGAAGGATTCCTATG-3′ and 3′-GGTCTAAACATGATCTGGG-5′). For transcriptomic analysis, RNA extracts quality was analyzed with Bioanalyzer 2100 (Agilent) and sequencing in triplicates was performed with NextSeq™ 500 (Illumina). RAW Data were analyzed and normalized using DESeq2 package [33]. Enrichment analysis were performed using GSEA Software 3.0 [34] and Enrichr webtool [35, 36].

### Western blotting

Whole cell extracts and western blotting were performed as previously described [37]. Antibodies against NCOA4 was from Bethyl, anti-FTH1, anti-LC3 were from Cell Signaling. Anti-CD71 antibody was purchased from Proteintech. anti-β-ACTIN was from Sigma-Aldrich.

### Measurement of synergistic effects

Cell viability was calculated for every dose combination of DHA and ferroptosis inducer using the Synergy Finder webtool (https://synergyfinder.fimm.fi/) and compared to each agent alone. Calculations were based on the ZIP model [24].

### Viability assay

AML cells were plated at 20 × 10^4^/mL in 100 μl of 10% FCS-supplemented RPMI prior to the addition of compounds. Cells were cultured in the presence of the test compounds for 24 to 48 h at 37 °C. Viability was quantified using the fluorescence based Uptiblue assay (Interchim, Montluçon, France). Briefly, Uptiblue was added to each well in 10 mL aliquots. Fluorescence was then measured with a Typhoon FLA9500 scanner (GE Healthcare; IL). Fluorescence values were normalized to dimethyl sulfoxide (DMSO)-treated controls for each AML cell line. Half maximal inhibitory concentration (IC^50^) values were calculated using a three parameter non-linear regression curve with Graph Pad Prism v9 (GraphPad, La Jolla, CA, USA). The same method was used for primary AML cells.

### Statistical analysis

All statistical calculations were performed using GraphPad Prism (version 9.0). Results are represented as mean ± SD. The differences between the two groups were performed by the Student’s t-test. Comparisons among multiple groups were analyzed by one-way ANOVA. Statistical significance was defined at *p < 0.05, **p < 0.01, ***p < 0.001 compared to the corresponding control.

**Table:** 

Reagent or resource	Source	Identifier
*Antibody*		
Ferritin Heavy Chain (FTH1), Rabbit Monoclonal	Cell Signaling	4393
LAMP2, Mouse Monoclonal	Cell Signaling	49067
NCOA4, Rabbit Monoclonal	Bethyl	A302-272A
LC3B, Rabbit monoclonal	Cell Signaling	49067
CD71	Proteintech	10084-2-AP
NRF2	Cell Signaling	12721
xCT	Cell Signaling	12691
*Compounds*		
RSL3	SelleckChem	S8155
FIN56	SelleckChem	S8254
Erastin	SelleckChem	S7242
Ferrostatin-1	SelleckChem	S7243
ZVAD-FMK	Thermofisher	Cat#S7023
Deferoxamine	SelleckChem	S5742
Deferasirox	SelleckChem	S1712
L -C-Propargylglycine (PPG)	Sigma	81838
Brusatol	Selleckchem	S7956
*Staining dyes*		
C11-BODIPY	Invitrogen	D3861
GSSG/GSH	Abcam	ab156681

## Supplementary information

Supplemental uncropped WB

Supplemental figure legends

Supplemental patient characteristics

Supplemental 1

Supplemental 2

Supplemental 3

Supplemental 4

Supplemental 5

Supplemental 6

Supplemental 7

Supplemental 8

Supplemental 9

Supplemental 10

Supplemental 11

## Data Availability

All data are available in the main text or the supplementary materials.
