# Supplementary figures and images for: Dihydroartemisinin-induced ferroptosis in acute myeloid leukemia: links to iron metabolism and metallothionein
Source: Cell Death Discov. 2023 Mar 17;9:97. doi: 10.1038/s41420-023-01371-8 (PMC10020442; doi:10.1038/s41420-023-01371-8)

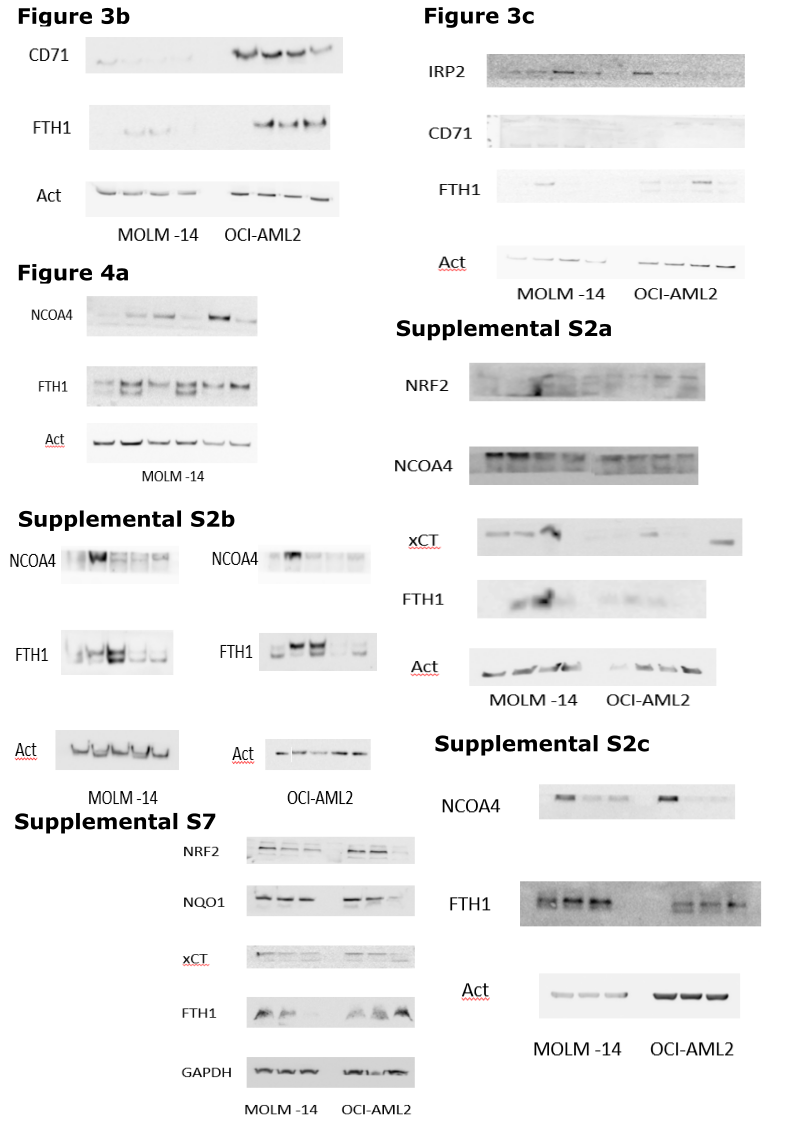

Supplement: Supplementary file 1 — Supplemental uncropped WB [file 41420_2023_1371_MOESM1_ESM.png]

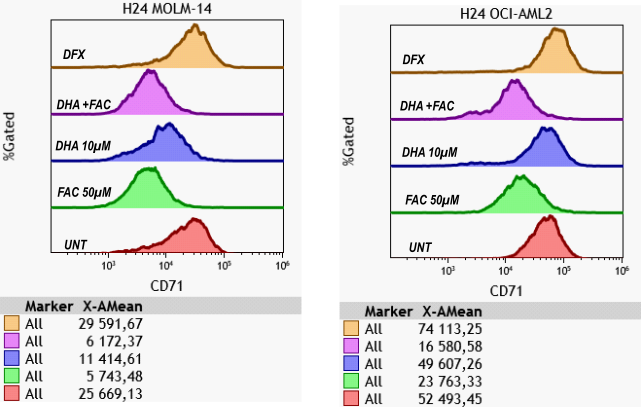

Supplement: Supplementary file 4 — Supplemental 1 [file 41420_2023_1371_MOESM4_ESM.png]

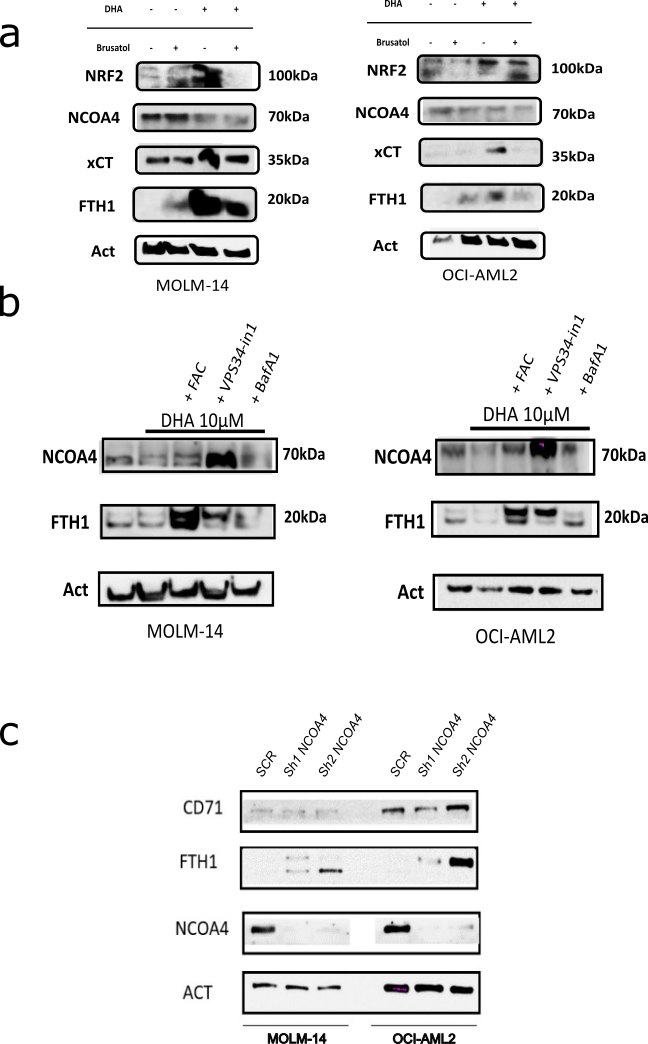

Supplement: Supplementary file 5 — Supplemental 2 [file 41420_2023_1371_MOESM5_ESM.png]

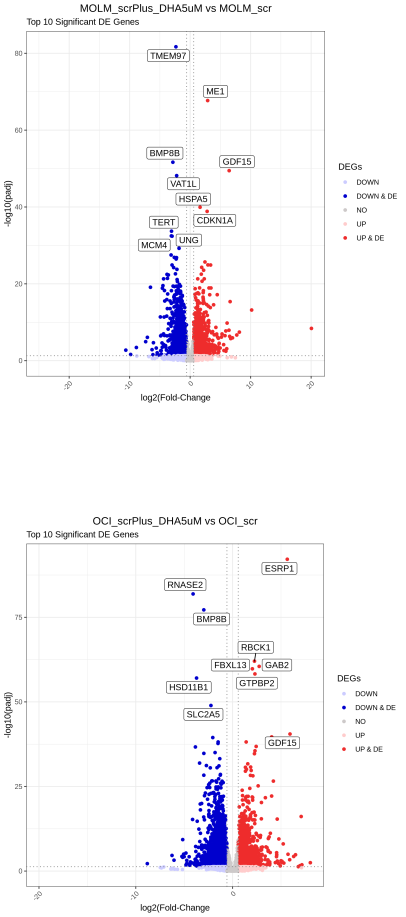

Supplement: Supplementary file 6 — Supplemental 3 [file 41420_2023_1371_MOESM6_ESM.png]

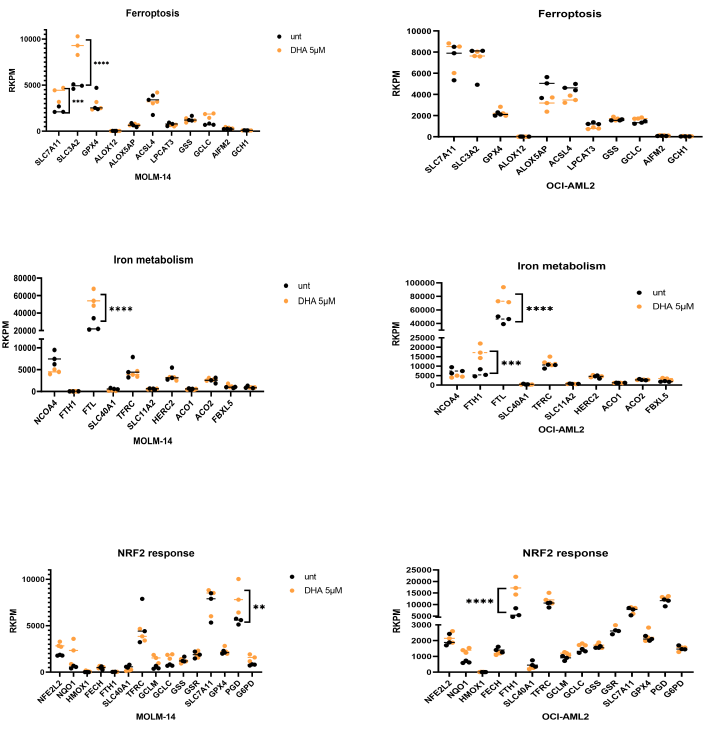

Supplement: Supplementary file 7 — Supplemental 4 [file 41420_2023_1371_MOESM7_ESM.png]

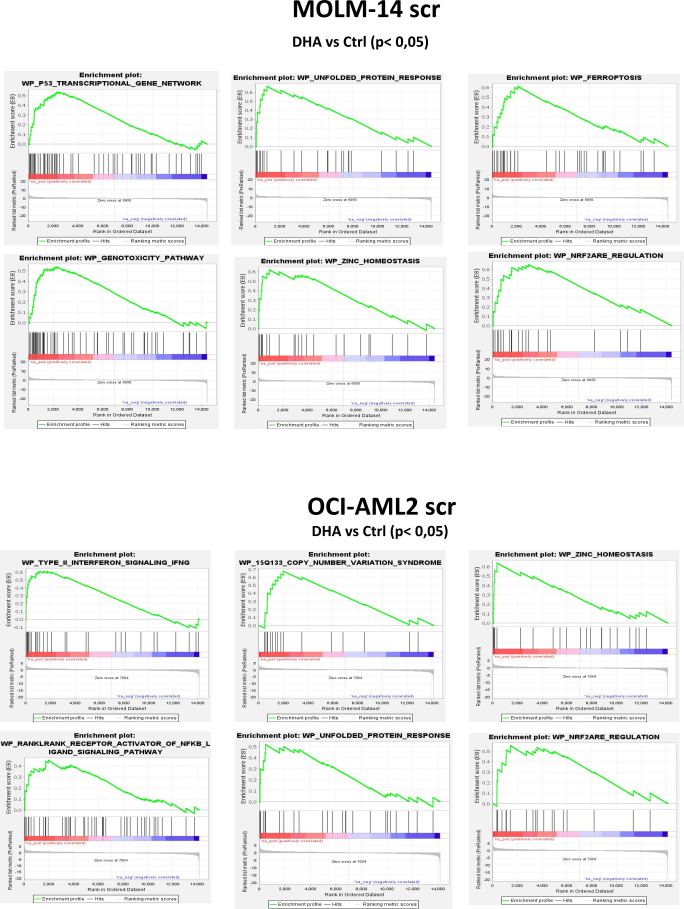

Supplement: Supplementary file 8 — Supplemental 5 [file 41420_2023_1371_MOESM8_ESM.png]

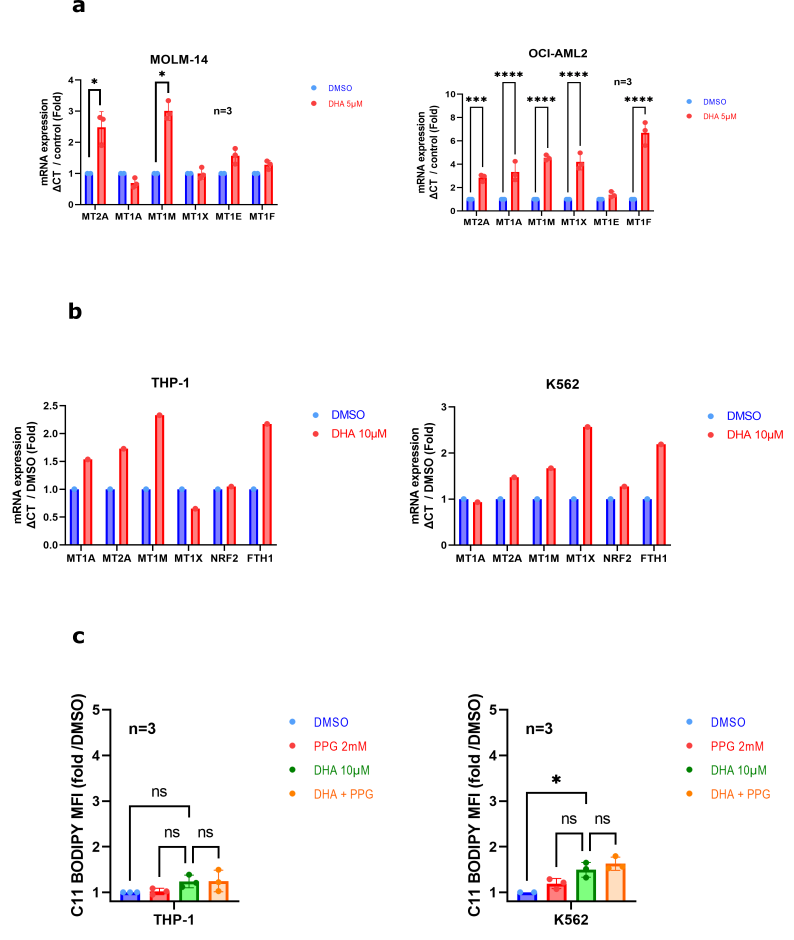

Supplement: Supplementary file 9 — Supplemental 6 [file 41420_2023_1371_MOESM9_ESM.png]

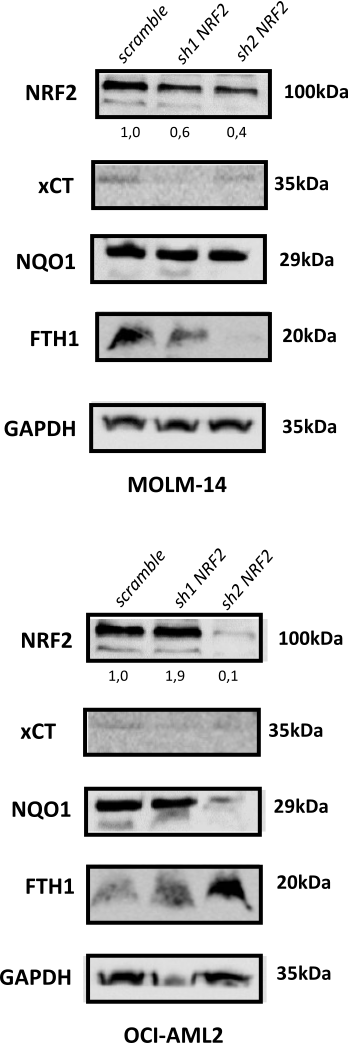

Supplement: Supplementary file 10 — Supplemental 7 [file 41420_2023_1371_MOESM10_ESM.png]

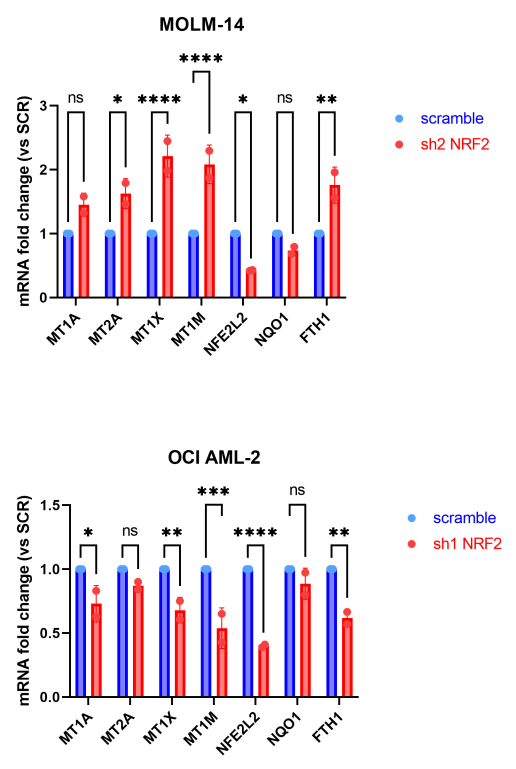

Supplement: Supplementary file 11 — Supplemental 8 [file 41420_2023_1371_MOESM11_ESM.png]

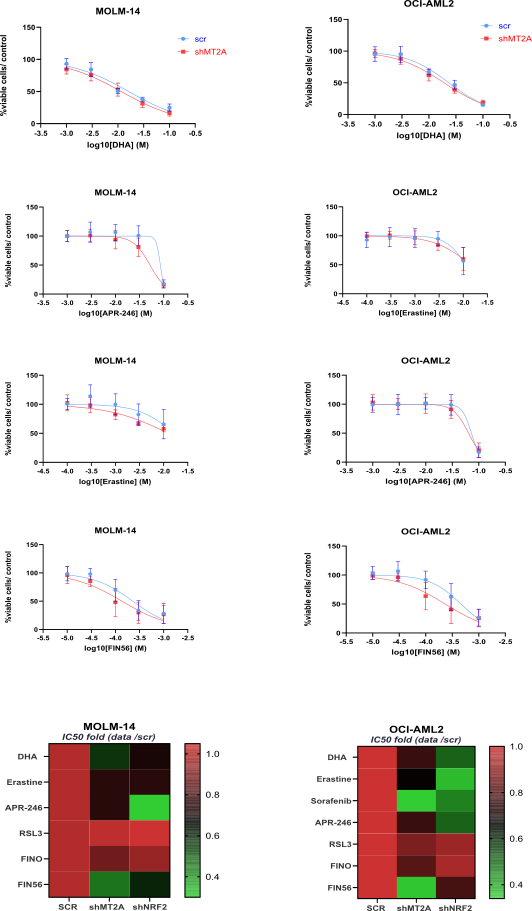

Supplement: Supplementary file 12 — Supplemental 9 [file 41420_2023_1371_MOESM12_ESM.png]

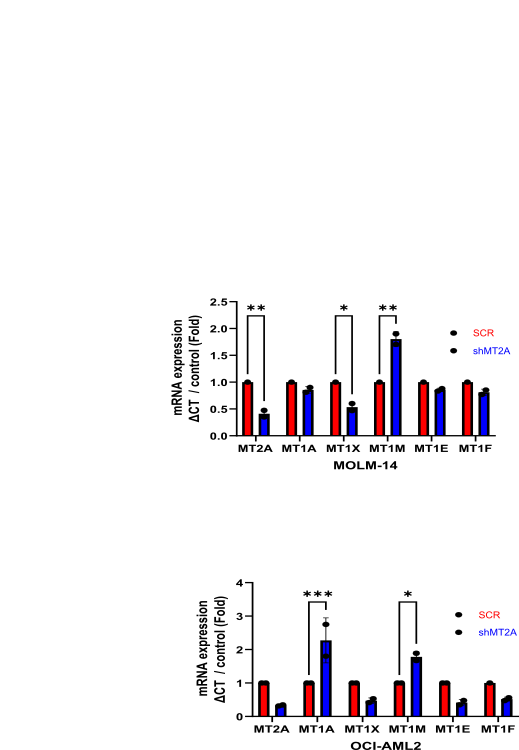

Supplement: Supplementary file 13 — Supplemental 10 [file 41420_2023_1371_MOESM13_ESM.png]

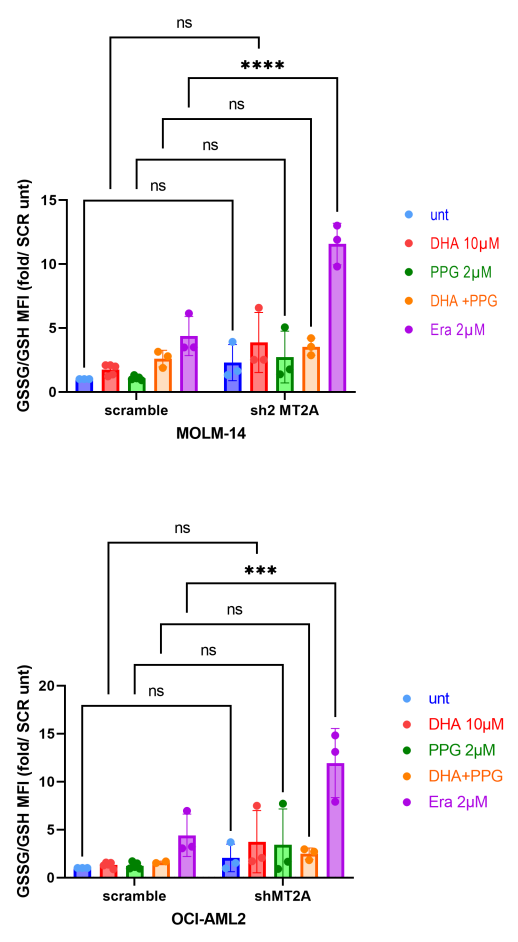

Supplement: Supplementary file 14 — Supplemental 11 [file 41420_2023_1371_MOESM14_ESM.png]
