# Supplementary material for: Dihydroartemisinin-induced ferroptosis in acute myeloid leukemia: links to iron metabolism and metallothionein
Source: Cell Death Discov. 2023 Mar 17;9:97. doi: 10.1038/s41420-023-01371-8 (PMC10020442; doi:10.1038/s41420-023-01371-8)
Supplement: Supplementary file 2 — Supplemental figure legends [file 41420_2023_1371_MOESM2_ESM.docx]

**Supplemental 1**. CD71 expression in MOLM-14 or OCI-AML2 cell lines after 24h exposure of indicated treatments and corresponding MFI (n=3).

**Supplemental 2.** A)Immunoblotting analysis of NRF2, NCOA4, xCT and FTH1 after DHA exposure (5 µM) for 6 hours, alone or with the NRF2 inhibitor brusatol (10 nM) (n=3). B) Immunoblotting analysis of NCOA4 and FTH1 after DHA exposure (10 µM) for 24 hours, alone or in association with FAC (50µM), VPS34-in1 (5µM) or bafilomycine (1µM) (n=3). C)Immunoblotting analysis of CD71, NCOA4 and FTH1 in MOLM-14 and OCI-AML2 cells transduced with shScramble, sh1 or sh2 NCOA4 and induced with doxycycline for 48h (n=3).

**Supplemental 3.** Fireplot showing the top-ten most up- and downregulated genes in the MOLM-14 shSCR and OCI-AML2 shSCR after 24 exposure with DHA (5µM) or without treatment, in transcriptomic analysis by RNAseq (Log2 FC>1,5; p<0,05).

**Supplemental 4.** Differential expression according to KEGG pathway 2021 of the main proteins between cells treated with DHA (5µM) for 24h, and control cells, for ferroptosis, iron metabolism and NRF2 pathways. *p<0,05; ** p<0,01; ***p< 0,001; ****p< 0,0001.

**Supplemental 5.** Six-top score enriched pathways according to GSEA analysis in either MOLM-14 SCR or OCI-AML2 SCR treated with DHA (5µM) for 24h versus control, p<0,05.

**Supplemental 6.** a) qPCR analysis of the six MT isoforms in MOLM-14 and OCI-AML2 after DHA treatment (5 µM) for 24 hours versus control (n=3). B) qPCR analysis of MT isoforms in THP-1 and K562 cell lines after 24h exposure with DHA (10µM) versus control (n=2). C) C11-BODIPY staining of THP-1 and K562 cells treated DHA (5µM) alone or in association with PPG (2mM). Datas are expressed in mean fluorescence intensity (MFI) (fold/ DMSO treated cells) (n=3).

**Supplemental 7.** Immunoblotting analysis of NRF2, xCT, NQO1 and FTH1 in MOLM-14 and OCI-AML2 cells transduced with shScramble, sh1 or sh2 NRF2 and induced with doxycycline for 48h (n=3).

**Supplemental 8.** qPCR analysis of four MT isoforms, NRF2 (NFE2L2) and NRF2 downstream target genes NQO1 and FTH1 in MOLM-14 and OCI-AML2 shScramble or sh2 NRF2 after 48h induction with doxycycline (n=2).

**Supplemental 9.** Viability curves for the indicated cells at 24 h post treatment. Error bars ± standard deviation are shown (n=6). (B) Heat maps showing IC_50_% of the indicated compounds after 24h of exposure in MOLM-14 and OCI-AML2 cells transduced with shMT2A vs shScramble (n=6).

**Supplemental 10.** qPCR analysis of the six MT isoforms in MOLM-14 and OCI-AML2 transduced with shScramble or shMT2A after 48h induction with doxycycline (n=2).

**Supplemental 11.** GSH/GSSG staining of MOLM-14 and OCI-AML2 cells transduced with shSCR or shMT2A for 48h and treated with indicated compounds for 24h. Datas are expressed in fold change compared to SCR non treated cells (n=3).

**Supplemental patient characteristics**

| Patient number | Age | FAB classification | Karyotype | Molecular abnormalities | De novo or secondary |
| --- | --- | --- | --- | --- | --- |
| #1 | 72 | AML 0 | normal | **NPM1 mutated**  *NGS* : NRAS, PTPN11, DNMT3A | De Novo |
| #2 | 79 | AML 1 | Del 7q | No mutations in NPM1, FLT3 or IDH | Secondary to MDS |
| #3 | 26 | NA | Del 17p | **TP53 mutated**  *NGS* : NRAS, PTPN11, MPL, KDM6 | De novo |
