# Supplementary material for: Dihydroartemisinin-induced ferroptosis in acute myeloid leukemia: links to iron metabolism and metallothionein
Source: Cell Death Discov. 2023 Mar 17;9:97. doi: 10.1038/s41420-023-01371-8 (PMC10020442; doi:10.1038/s41420-023-01371-8)
Supplement: Supplementary file 3 — Supplemental patient characteristics [file 41420_2023_1371_MOESM3_ESM.pdf]

PATIENT NUMBER	AGE	FAB CLASSIFICATION	KARYOTYPE	MOLECULAR ABNORMALITIES	DE NOVO OR SECONDARY
**#1**	72	AML 0	normal	**NPM1 mutated**<br>*NGS* : NRAS, PTPN11, DNMT3A	De Novo
**#2**	79	AML 1	Del 7q	No mutations in NPM1, FLT3 or IDH	Secondary to MDS
**#3**	26	NA	Del 17p	**TP53 mutated**<br>*NGS* : NRAS, PTPN11, MPL, KDM6	De novo
